# Phylloquinone supplementation improves glycemic status independent of the effects of adiponectin levels in premonopause women with prediabetes: a double-blind randomized controlled clinical trial

**DOI:** 10.1186/s40200-014-0127-9

**Published:** 2015-01-14

**Authors:** Hamid Rasekhi, Majid Karandish, Mohammad-Taha Jalali, Majid Mohammadshahi, Mehdi Zarei, Azadeh Saki, Hajieh Shahbazian

**Affiliations:** Nutrition and Metabolic Diseases Research Center, Ahvaz Jundishapur University of Medical Sciences, Ahvaz, Iran; Hyperlipidemia Research Center, Ahvaz Jundishapur University of Medical Sciences, Ahvaz, Iran; Department of Food Hygiene, Faculty of Veterinary Medicine, Shahid Chamran University of Ahvaz, Ahvaz, Iran; Department of Biostatistics and Epidemiology, School of Health, Ahvaz Jundishapur University of Medical Sciences, Ahvaz, Iran; Diabetes Research Center, Ahvaz Jundishapur University of Medical Sciences, Ahvaz, Iran

**Keywords:** Phylloquinone, Vitamin K1, Osteocalcin, Adiponectin, Prediabetes

## Abstract

**Background:**

Vitamin K, as a cofactor in the gamma carboxylation of certain glutamic acid (Gla) residues, has been related to glucose metabolism and insulin sensitivity. Osteocalcin, also known as bone γ-carboxyglutamic acid, increases β-cell proliferation as well as insulin and adiponectin secretion, which improve glucose tolerance and insulin sensitivity. Thus, the purpose of the present study was to examine the possible role of adiponectin as a mediator of glucose homeostasis following phylloquinone supplementation in premonopause women with prediabetes.

**Methods:**

Eighty two women were randomized to consume vitamin k1 supplement (n = 39) or placebo (n = 43) for four weeks. Participants in vitamin K1 treatment group received one pearl softgel capsule containing 1000 micrograms phylloquinone while the placebo group received one placebo capsules daily for four weeks. The Blood samples were collected at baseline and after a four-week intervention to quantify osteocalcin, adiponectin, leptin and relevant variables.

**Results:**

Phylloquinone supplementation significantly increased serum adiponectin concentration (1.24 ± 1.90 compared with −0.27 ± 1.08 μg/ml), and did not alter total osteocalcin (0.50 ± 4.11 compared with 0.13 ± 1.85 ng/ml) and leptin (−0.29 ± 8.23 compared with −1.15 ± 5.25 ng/ml) compared with placebo. Adjustments for total osteocalcin and adiponectin using analysis of covariance (ANCOVA) did not affect the association of glycemic status with related variables.

**Conclusions:**

In conclusion our study demonstrated that phylloquinone supplementation improved glycemic status in premonopausal prediabetic women independent of adiponectin.

**Trial registration:**

This trial was registered in Iranian Registry of Clinical Trials with ID number of IRCT2013120915724N1.

## Background

Vitamin K is a fat-soluble vitamin that functions as a cofactor in the gamma carboxylation of certain glutamic acid (Gla) residues of vitamin K dependent proteins such as osteocalcin [1]. Studies in animal models showed osteocalcin (OC), an extracellular matrix component secreted by osteoblast in the bone, to be the mediator of energy metabolism in bone, pancreas and adipose tissue. In addition, OC in its undercarboxylated form (ucOC) also enhanced insulin sensitivity by up-regulating adiponectin expression in adipose tissues [2,3]. Also, Circulating undercarboxylated osteocalcin is an index of the vitamin K status decreases in response to vitamin K supplementation [4]. Several epidemiological studies have examined the association of vitamin K intake and glycemic status and insulin homeostasis. Higher intake of vitamin k also vitamin k supplementation has been associated to reduction in insulin resistance and improved glycemic status [5-10]. These findings are in contrast to some other studies which, based on animal models, anticipate reduction in ucOC levels by increasing vitamin k intake and hence results in adverse effects on glycemic status. Therefore, it is likely that vitamin K exerts its influence on glycemic status through other mechanisms.Therefore, in this study the effect of vitamin K supplementation on osteocalcin and adiponectin and consequently their impact on glucose and insulin homeostasis was investigated.

## Methods

### Subjects

A randomized, double-blinded, placebo controlled clinical trial was designed conducted over a total period of four weeks. This clinical trial was approved by the Ethics Committee of Ahvaz Jundishapur University of Medical Sciences. Written informed consent was obtained from all study participants. Premenopausal women with diagnosed prediabetes with ages 22 to 45 years and body mass index (BMI) of 18.5 to 30 kg/m2 were eligible for the study. Subjects were recruited based on the results of a standard 75-g Oral Glucose Tolerance Test (OGTT) at screening (blood drawn in the 0 and 120 min), according to the American Diabetes Association criteria [11]: Predibetes (PDM) was diagnosed according to the criteria established by the American Diabetes Association, i.e., impaired fasting glucose (IFG) (100 < fasting plasma glucose (FPG) < 126) or impaired Glucose test (IGT) (140 < glucose120 min <200).

A total of 82 prediabetes women met the inclusion criteria. None of the subjects suffered rheumatic, thyroid, parathyroid, kidney, or liver disease, pregnancy, lactation, menopause and drugs known to influence glucose, vitamin k and bone metabolism like insulin and glucose, lipid-lowering drugs, warfarin, corticosteroids, vitamin and mineral supplements within six months. Overall, 82 women were randomized to consume vitamin k1supplement (n = 39) or placebo (n = 43) for four weeks. Participants in vitamin K1 treatment group received one pearl softgel capsule containing 1000 micrograms phylloquinone (DSM Nutritional Products, Inc, Switzerland) while the placebo group received one placebo capsules (Barij Essence Co. Iran) daily for four weeks. Placebo capsules were similar in color, shape, size appearance, and packaging and were indistinguishable for participants and investigators. The participants were asked to maintain their habitual food consumption, body weight and physical activity pattern throughout the study and not to consume any supplements other than the one provided to them by the investigator. Dietary intake was assessed using a three-day food record consisting of three non-consecutive days, including two week days and one weekend day. The dietary records were based on estimated values in household measurements. To obtain nutrient intakes of participants on the basis of these 3-d food diaries, we used Nutritionist IV software (First Databank) modified for Iranian foods. The short form of IPAQ consists of seven questions assessing the frequency and duration of participation in vigorous, moderate and walking activity and the time spent sitting during the last week was used to determine physical activity levels [12]. The Persian translation of this questionnaire has previously been validated [13].

### Assessment of variables

Body mass index and body fat were measured before and after the intervention. Body weight was measured to the nearest 0.1 kg after overnight fasting, without shoes and wearing minimal clothing, by the use of a digital scale (Seca). Height was measured to the nearest 0.1 cm by using a non-stretched tape measure (Seca). BMI was calculated as weight in kilograms divided by height in meters squared (kg/m^2^*)*. The body fat was measured by OMRON BF 306 (Dalian, China) body fat monitor using bioelectric impedance. The Blood samples (10 ml) were collected at baseline and after a four-week intervention. After an overnight fast, 75 g OGTT was given to each subject between 8 a.m. and 10 a.m. Plasma glucose was determined at times 0 and 120 min after OGTT on the same day. Then the remaining separated serum stored at −70 C before analysis in the laboratory of Ahvaz Jundishapur University of Medical Sciences. Serum carboxylated and undercarboxylated osteocalcin (cOC and ucOC) were measured blood serum using Takara Bio Inc., EIA kits (MK118 and MK111, respectively, Bio Inc., Japan,). Total OC (tOC) was estimated as the sum of cOC and ucOC [14]. Adiponectin and leptin were quantitatively determined using a commercially available enzyme immunoassay (EIA) kit from Boster (china). Serum insulin (INS) was assayed by using an ELISA kit (Q-1-DIAPLUS, USA); Fasting blood glucose (FBG) and 2-h post-OGTT glucose were measured by auto-analyser (Hitachi, USA); hemoglobin A1C (HbA1c) was measured with HLC-723G8 (Tosoh Co., Tokyo, Japan) by high performance liquid chromatography (HPLC). Insulin resistance was calculated with HOMA-IR, which was defined as: HOMA-IR = [FPS (mg/DL)*fasting insulin (FINS (μU/ml)]/405. Basal insulin secretion which was calculated by using the following formula HOMA-%B:360*FINS(μU/ml)/(FPG-63) [15]. Quantitative insulin sensitivity check index (QUICKI) were calculated on the basis of suggested formulas: 1/[log (Insulin μU/ml) + log (Glucose mg/DL)] [16].

### Statistical analyses

Data are expressed as means ± SDs. The normality of data distribution was assessed by using the Kolmogorov-Smirnov goodness-of-fit test. Two-factor repeated-measures analysis of variance (ANOVA) was used to test time*group interactions, with time and treatment as factors. In case of a significant time*group interaction, a between-group comparison of changes at 4 weeks was done by Independent-samples Student’s t test analysis. When the time effect was significant, the within-group comparison of values was performed by the paired-samples t test. Differences in proportions were evaluated by using a chi-square test. ANCOVA was performed to examine the association between supplementation group and measure of insulin sensitivity and glucose. All statistical analyses were done by using the Statistical Package for Social Sciences (SPSS version 16; SPSS Inc, Chicago, IL). The P < 0.05 was considered significant.

## Results and discussion

### Results

The means and standard deviations for age, weight, and BMI were 40.17 ± 4.9 years, 71 ± 6.5 kg, and 28.08 ± 1.65 *kg*/*m*^*2*^, respectively. Most of the women were overweight and BMI distribution was not significantly different between two groups. According to the IPAQ questionnaire, the both groups had the same low level of physical activity. Table 1 shows the baseline and end-of-trial characteristics of the participants. Table 2 shows their dietary intakes of relevant nutrients. No significant difference existed between phylloquinone and placebo groups. Baseline values of total osteocalcin, adiponectin, leptin, FBS, 2-h post-OGTT glucose, fasting and 2-h post-OGTT insulin have been shown in Table 3. The time effects were statistically significant on adiponectin, FBS, 2-h post-OGTT glucose and 2-h post-OGTT insulin. Also the time*group interaction effects on adiponectin and 2-h post-OGTT glucose were significant (all P, 0.001). Intake of phylloquinone supplement led to significant decrease in 2 h post-OGTT glucose (−10.87 ± 27.41 compared with 1.20 ± 18.63 mg/dl), 2 h post-OGTT insulin level (−17.46 ± 44.97 compared with 5.88 ± 23.65 μIU/ml) and a significant increase in serum adiponectin concentration (1.24 ± 1.90 compared with −0.27 ± 1.08 μg/ml) compared with placebo. We did not find any significant effect of phylloquinone supplementation on total osteocalcin, leptin and related glycemic and insulin variables. To evaluate the effect of each of the total osteocalcin and adiponectin on the changes of glucose and insulin levels between groups, we adjusted them for tOC and adiponectin (Table 4).

**Table 1:** **General characteristics of premenopausal women with PDM who received either vitamin K1 supplements or placebo**

	**Placebo group (n = 43)**	**Phylloquinone group (n = 39)**	**P value**
Age (y)	40.09 ± 4.65	40.25 ± 5.32	0.88*
Weight (kg) at study baseline	71.09 ± 6.59	71.21 ± 6.47	0.93*
Weight (kg) at end of trial	71 ± 6.76	70.72 ± 6.39	0.84*
BMI (18.5-24.9 kg/m^2^) at study baseline	1(2.3%)	3(7.7%)	0.342**
BMI (18.5-24.9 kg/m^2^) at the end of trial	2(4.7%)	5(12.8%)	0.249**
BMI (25–29.9 kg/m^2^) ) at study baseline	42(97.7%)	36(92.3%)	0.342**
BMI (25–29.9 kg/m^2^) at the end of trial	41(95.3%)	34(87.2%)	0.249**
FM (%) at study baseline	38.55 ± 3.99	38.77 ± 3.86	0.79*
FM (%) at end of trial	38.57 ± 4.10	38.46 ± 4.05	0.90*
HbA_1_C (%) at study baseline	5.91 ± 0.49	5.81 ± 0.64	0.42*

All values are means ± SDs.

*Obtained from independent-samples t test.

**Obtained from chi-square test.

**Table 2:** **Dietary intakes of prediabetic women who received either vitamin K1 supplements or placebo throughout the study**

	**Placebo group (n = 43)**	**Vitamin K1 group (n = 39)**	**P value** ^**1**^
Energy (kcal/d)	1769 ± 275	1819 ± 272	0.42
Carbohydrate (g/d)	243.12 ± 37.76	249.60 ± 39.20	0.44
Protein (g/d)	75.04 ± 12.75	77.56 ± 11.60	0.35
Fat (g/d)	64.90 ± 10.05	66.32 ± 10.34	0.52
Vitamin k (μg/d)	57.16 ± 19.03	62.69 ± 15.45	0.15
Vitamin D (mg/d)	3.7 ± 1.6	4.06 ± 1.67	0.32
Calcium (mg/d)	719.38 ± 261	677.0 ± 237.97	0.44

All values are means ± SDs.

^1^Obtained from independent-samples t test.

**Table 3:** **Metabolic variables, biomarkers of insulin resistance and comparison of changes within and between placebo and phylloquinone groups**

	**Placebo group (n = 43)**			**Phylloquinone group (n = 39)**			
	**Week0**	**Week4**	**Change**	**Week0**	**Week4**	**Change**	**P value** ^**1**^
Total Osteocalcin (ng/ml)	11.95 ± 5.76	12.08 ± 5.65	0.13 ± 1.85	14.50 ± 5.81	15.01 ± 6.51	0.50 ± 4.11	0.61
leptin (ng/ml)	26.78 ± 10.33	25.62 ± 10.21	−1.15 ± 5.25	28.59 ± 9.61	28.29 ± 9.86	−0.29 ± 8.23	0.92
Adiponectin (μg/ml)	8.81 ± 1.54	8.54 ± 1.87	−0.27 ± 1.08	9.19 ± 1.80	10.44 ± 1.20*	1.24 ± 1.90	0.00
FBS (mg/DL)	106.69 ± 13.63	105.20 ± 12.41	−1.48 ± 10.37	107.66 ± 10.72	104 ± 11.25*	−3.12 ± 9.22	0.45
2-h-post-OGTT glucose (mg/DL)	155.27 ± 15.97	156.48 ± 26.44	1.20 ± 18.63	143.84 ± 35.30	132.97 ± 27.37*	−10.87 ± 27.41	0.02
FINS (μIU/ml)	23.83 ± 12.08	23.80 ± 8.28	−0.02 ± 8.92	20.74 ± 9.90	20.56 ± 8.04	−0.18 ± 5.92	0.92
2-h-post-OGTT INS (μIU/ml)	106.55 ± 46.96	112.43 ± 53.19	5.88 ± 23.65	97.80 ± 54.50	80.34 ± 42.24*	−17.46 ± 44.97	0.00

All values are means ± SDs. *Different from week 0, P < 0.05; OGTT, oral glucose tolerance test; INS, Insulin; FINS, Fasting insulin; FBS, Fasting blood sugar.

^1^Obtained from independent-samples t test.

**Table 4:** **Adjusted changes in metabolic variables in prediabetic women who received either vitamin K1 supplements or placebo**

	**Placebo group (n = 43)**	**Vitamin K1 group (n = 39)**	**P value** ^**1**^
FBS (mg/DL)			
Model1	1.48 ± 1.50	−3.12 ± 1.57	0.45
Model2	−1.46 ± 1.51	−3.13 ± 1.58	0.45
Model3	−1.97 ± 1.59	−2.59 ± 1.67	0.80
2-h post-OGTT glucose (mg/DL)			
Model1	1.20 ± 3.54	−10.87 ± 3.71	0.02
Model2	1.36 ± 3.54	−11.04 ± 3.71	0.01
Model3	2.48 ± 3.74	−12.28 ± 3.95	0.01
FINS (μIU/ml)			
Model1	−0.02 ± 1.16	−0.18 ± 1.22	0.92
Model2	0.003 ± 1.17	−0.21 ± 1.23	0.89
Model3	-.039 ± 1.24	−0.16 ± 1.31	0.94
2-h post-OGTT insulin (μIU/ml)			
Model1	5.88 ± 5.40	−17.46 ± 5.67	0.00
Model2	5.69 ± 5.41	−17.25 ± 5.60	0.00
Model3	9.90 ± 5.58	−21.88 ± 5.89	0.00
HOMA-IR			
Model1	−0.05 ± 0.32	−0.22 ± .34	0.76
Model2	-.05 ± 0.32	−0.22 ± 0.34	0.71
Model3	−0.06 ± 0.34	−0.20 ± 0.36	0.79
HOMA-B			
Model1	−5.42 ± 14.49	14.95 ± 15.21	0.33
Model2	4.84 ± 14.50	14.31 ± 15.23	0.36
Model3	−7.10 ± 15.42	16.80 ± 16.28	0.31
QUICKI			
Model1	−0.004 ± 0.002	−0.001 ± 0.002	0.43
Model2	−0.004 ± 0.002	−0.001 ± 0.002	0.44
Model3	−0.004 ± 0.002	−0.00 ± 0.003	0.33

All values are means ± SEs. Model 1 show original raw data**,** Model 2 was adjusted for tOC; Model 3 was adjusted for adiponectin; tOC, Total osteocalcin; FBS, Fasting blood sugar; OGTT, Oral glucose tolerance test; FINS, Fasting insulin; HOMA-IR, Homeostasis model assessment insulin resistance index; HOMA-B, Homeostatic model assessment–β cell function; QUICKI, Quantitative insulin sensitivity check index. ^1^Obtained from ANCOVA.

### Discussions

Several epidemiological studies have investigated the association between vitamin K intake and glycemic status. For instance, in a cross-sectional study of 2719 adults 26–81 years of age, Yoshida et al. [7] showed that higher intake of vitamin K was associated with better insulin sensitivity and lower post-challenge glucose levels. In addition, Beulens JW et al. [5], found that higher intakes of vitamin K were associated with reduced risk of type 2 diabetes. In another study with similar results it was reported in a National Health and Nutrition Examination Survey (NHANES) 1999–2004 that vitamin K intake in the highest, compared with the lowest, quintile was associated with lower prevalence of hyperglycemia as a component of metabolic syndrome [6]. However, the exact mechanisms underlying the associations between vitamin k intakes, glycemic status and insulin homeostasis still remain unknown. One finding which could possibly help shed light on the mechanism has been reported by Lee et al. [3]. They demonstrated that osteocalcin, a vitamin-K-dependent protein in bone, is involved in glucose metabolism by increasing insulin secretion and cell proliferation in pancreatic β-cells and by upregulating the expression of the adiponectin gene in adipocytes, thus improving insulin sensitivity. This study was devised as a first study of its kind, to explore the impacts of the carboxylation of Osteocalcin by vitamin K and the mediation effects of adiponectin in prediabetic, premonopause women. In this study, it was observed that phylloquinone supplementation for four weeks significantly increased serum adiponectin, decreased 2-h post-OGTT glucose and did not change the other related variables compared with placebo. In contrast, Knapen MH [17] found that Supplementation with vitamin K did not affect circulating adiponectin concentrations. Choi et al. [18] also reported that no significant alterations were seen in fasting plasma glucose and adiponectin concentrations in serum with 4 weeks Vitamin K2 Supplementation in healthy young male subjects. Cross sectional studies have also pointed to the relationships between these variables. Kanazawa et al. [19] found that that ucOC/OC ratio positively correlated with serum adiponectin level in men. Reinehr T [20] studied obese children and found no significant relationship between adiponectin and osteocalcin. Meanwhile, another study failed to show such an association between adiponectin and osteocalcin [21].

In the current study, analysis of covariance was performed to determine the role of either osteocalcin or adiponectin in glucose and insulin homeostasis. It was observed that adjustments for total steocalcin and adiponectin did not alter the associations of the related variables to glycemic status. Along the same lines, Shea et al. [22] reported that the strength of the association between total osteocalcin and carboxylated osteocalcin with HOMA-IR was somewhat attenuated after adiponectin was accounted for; therefore, they concluded that the association between total osteocalcin and carboxylated osteocalcin with HOMA-IR may depend partially on adiponectin. Chen X et al. [23] reported that the negative association between HOMA-IR and tOC remained significant after being controlled for adiponectin. In Hwang YC et al. study [24] on adult subjects, it was reported that although the circulating osteocalcin level was associated with improved glucose tolerance and insulin secretion, this was independent of the plasma adiponectin level.

Although these studies and their contradictory results have considered the relationships between adiponectin and osteocalcin, it seems vitamin K probably influence Glycemic status through other mechanisms.

## Conclusions

Finally although this study could not provide the underlying mechanism we speculate that vitamin K1 supplementation could modulate glycemic status by mechanism other total osteocalcin and adiponectin in premonopausal prediabetes women. In conclusion our study demonstrated that phylloquinone supplementation improved glycemic status in premenopausal prediabetic women independent of adiponectin.
